# 4,4′-Bipyridine–butane-1,2,3,4-tetra­carboxylic acid (1/1)

**DOI:** 10.1107/S1600536808011732

**Published:** 2008-05-03

**Authors:** M. Mahdi Najafpour, Małgorzata Hołyńska, Tadeusz Lis

**Affiliations:** a Dorna Institute of Science, No. 83 Padadshahr, 14 St. Ahwaz, Khozestan, Iran; b Faculty of Chemistry, University of Wrocław, 14 Joliot-Curie St, 50-383 Wrocław, Poland

## Abstract

The title compound, C_10_H_8_N_2_·C_8_H_10_O_8_, is an example of a system with a short O⋯H⋯N hydrogen bond [O⋯N = 2.565 (3) Å]. The crystal structure comprises a 1:1 adduct between 4,4′-bipyridine and butane-1,2,3,4-tetra­carboxylic acid, where both components are centrosymmetric. The component mol­ecules are linked through strong O⋯H⋯N hydrogen bonds, forming chains extending approximately along [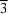
11]. The chains are inter­connected by O⋯H⋯O hydrogen bonds and weak stacking inter­actions involving the pyridyl rings of the 4,4′-bipyridine mol­ecules [centroid–centroid distance = 3.73 (2) Å and inter­planar distance = 3.35 (1) Å]. The H atom of the short O⋯H⋯N hydrogen bond is disordered over two positions with site occupancy factors of *ca* 0.6 and 0.4. One methylene group is disordered over two positions; the site occupancy factors are *ca* 0.9 and 0.1.

## Related literature

For related literature, see: Barnes & Barnes (1996 ▶); Cowan *et al.* (2003 ▶); Etter *et al.* (1990 ▶); Majerz *et al.* (1997 ▶); McKee & Najafpour (2007 ▶); Steiner *et al.* (2000 ▶, 2001 ▶); Wang & Chen (2005 ▶); Wang & Wei (2006 ▶).
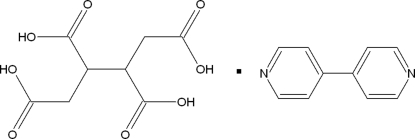


## Experimental

### 

#### Crystal data

C_10_H_8_N_2_·C_8_H_10_O_8_
*M* *_r_* = 390.34
Triclinic, 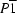

*a* = 5.642 (4) Å
*b* = 6.966 (4) Å
*c* = 11.680 (8) Å
α = 73.55 (5)°
β = 81.34 (5)°
γ = 73.85 (5)°
*V* = 421.6 (5) Å^3^
*Z* = 1
Mo *K*α radiation
μ = 0.12 mm^−1^
*T* = 100 (2) K
0.40 × 0.18 × 0.04 mm

#### Data collection

Oxford Diffraction KM-4-CCD diffractometer
Absorption correction: none
3650 measured reflections
1946 independent reflections
1034 reflections with *I* > 2σ(*I*)
*R*_int_ = 0.030

#### Refinement

*R*[*F* ^2^ > 2σ(*F* ^2^)] = 0.045
*wR*(*F* ^2^) = 0.083
*S* = 1.01
1946 reflections
135 parameters
2 restraints
H-atom parameters constrained
Δρ_max_ = 0.30 e Å^−3^
Δρ_min_ = −0.19 e Å^−3^

### 

Data collection: *CrysAlis CCD* (Oxford Diffraction, 2006 ▶); cell refinement: *CrysAlis RED* (Oxford Diffraction, 2006 ▶); data reduction: *CrysAlis RED*; program(s) used to solve structure: *SHELXS97* (Sheldrick, 2008 ▶); program(s) used to refine structure: *SHELXL97* (Sheldrick, 2008 ▶); molecular graphics: *SHELXTL* (Sheldrick, 2008 ▶); software used to prepare material for publication: *SHELXL97*.

## Supplementary Material

Crystal structure: contains datablocks global, I. DOI: 10.1107/S1600536808011732/tk2261sup1.cif

Structure factors: contains datablocks I. DOI: 10.1107/S1600536808011732/tk2261Isup2.hkl

Additional supplementary materials:  crystallographic information; 3D view; checkCIF report

## Figures and Tables

**Table 1:** Hydrogen-bond geometry (Å, °)

*D*—H⋯*A*	*D*—H	H⋯*A*	*D*⋯*A*	*D*—H⋯*A*
O11—H1*A*⋯N1	0.84	1.73	2.565 (3)	173
N1—H1*B*⋯O11	0.88	1.69	2.565 (3)	177
O12—H12⋯O21^iii^	0.84	1.91	2.747 (3)	175

Symmetry code: (iii) 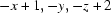.

**Fig. 1.**
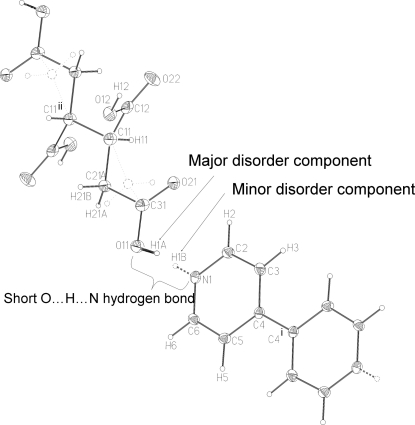
Component molecules showing atom labelling scheme and displacement ellipsoids at the 30% probability level. Symmetry codes: [i] -x + 3, -y, -z + 1; [ii] -x, -y + 1, -z + 2; . The minor component of the disordered BTCA molecule is shown as dotted lines. The minor component of the disordered H atom is shown with a dashed bond.

**Fig. 2.**
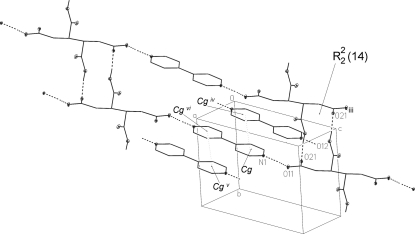
Chains formed by 4,4'-bipyridine and butane-1,2,3,4-tetracarboxylic acid molecules approximately along [311]. The hydrogen bonds are shown as dashed lines. The stacking interaction between the 4,4'-bipyridine molecules from the neighbouring chains is denoted with dotted line. The centroids of the interacting rings are denoted with the Cg symbol. Graph symbol (Etter et al., 1990) is given for the described ring motif. Symmetry codes: [iii] -x + 1, -y, -z + 2; [iv] -x + 2, -y, -z + 1; [v] x + 1, y, z; [vi] -x + 3, -y, -z + 1. The disordered part of the BTCA acid molecule as well as H atoms not involved in hydrogen bonds are omitted for clarity.

## supplementary crystallographic information

### Comment

Systems with short O···H···N hydrogen bonds have been widely studied. A correlation of the geometric parameters defining the O···H···N bridge for amine - phenol complexes and the pK_a_ values has been established (Majerz *et al.*, 1997 and references therein). It was shown that the shortest O···N distances of about 2.52 Å are realised when the proton is near the centre of the O···H···N bridge. The first example of a crystal structure of this type to be investigated using neutron diffraction was the adduct of 2–methylpyridine and pentachlorophenol (Steiner *et al.*, 2000). Also, temperature-dependent neutron diffraction studies have been performed, for example, on the 1:2 co–crystal of benzene-1,2,4,5-tetracarboxylic acid and 4,4'-bipyridine (Cowan *et al.*, 2003). One of the shortest known O···H···N hydrogen bonds was observed in the crystal structure of 4–methylpyridine and pentachlorophenol (Steiner *et al.*, 2001) with the O···N distance of 2.506 (3) Å at 20 K and the H atom essentially at the centre of the O and N atoms.

The title crystal is an example of a system with short O···H···N hydrogen bonds with the O···N distance being 2.565 (3) Å. It contains butane-1,2,3,4-tetracarboxylic acid (BTCA), which has been widely used as a cross-linking agent for cotton fabrics (Wang & Chen, 2005) and also in crystal engineering studies of hydrogen bonding arrays (Barnes & Barnes, 1996). Attempts to obtain the acid in crystalline form have so far been unsuccessful (Barnes & Barnes, 1996).

The crystal structure comprises 4,4'-bipyridine and butane-1,2,3,4-tetracarboxylic acid in a 1:1 ratio (Fig. 1 & Table 1). Both components are centrosymmetric. The carboxylic acid groups with C31 and C12 atoms are *gauche* with the C12—C11—C21A—C31 torsion angle being -48.6 (3)°. These groups are mutually twisted with the interplanar angle between the planes defined by O12, O22, C12, C11 and O11, O21, C31, C21A, respectively, being 73.7 (1)°.

There are only two reports of crystal structures containing anions of butane-1,2,3,4-tetracarboxylic acid: the structure of its ammonium (Barnes & Barnes, 1996) and guanidinium (McKee & Najafpour, 2007) salts. In both of these structures, the anions are centrosymmetric and not protonated. However, the conformation of the anion resembles that reported here with the torsion angles equivalent to C31—C21A—C11—C12 being -61.1 (2)° in the guanidinium salt (McKee & Najafpour, 2007), and -55.2 (2)° and -60.9 (2)° for the two symmetry independent anions in the ammonium salt (Barnes & Barnes, 1996).

The centrosymmetric 4,4'-bipyridine molecule is planar and its geometric parameters are comparable to other reported cases of planar molecule of this formula (*e.g.* Wang & Wei, 2006).

The butane-1,2,3,4-tetracarboxylic acid and 4,4'-bipyridine molecules are connected by short O···H···N hydrogen bonds (with H atom disordered over two positions - nearer to the O and nearer to the N atom with the occupancy factors of 0.59 (3) and 0.41, respectively) to form chains extending approximately along [311] (Fig. 2). In each such hydrogen bond the O···N distance is 2.565 (3) Å (Table 2). The chains are interconnected by the O—H···O hydrogen bonds where the O12 atom from one of the carboxylic groups participates as the donor and the O21 atom from the other carboxylic group as acceptor (Table 2). Thus, *R*^2^_2_(14) motifs are formed (Etter *et al.*, 1990). The chains also interact through weak stacking interactions between the pyridyl rings (Fig. 2) with the distance between the rings centroids of 3.73 (2) Å. The interplanar distance between the planes of interacting rings is 3.35 (1) Å.

### Experimental

4,4'-Bipyridine and butane-1,2,3,4-tetracarboxylic acid were purchased from Merck. A solution of butane-1,2,3,4-tetracarboxylic acid (1.5 mmol) in hot water (250 ml) was added dropwise to a vigorously stirred suspension of 4,4'-bipyridine (2.5 mmol) in water (25 ml) over a period of 5 min. and was heated to obtain a homogeneous solution. The solution was slowly cooled to room temperature. The resulting crystals in form of colourless plates were filtered and recrystallized from water.

The presence of short O···H···N hydrogen bond is confirmed by the IR spectrum collected in KBr pellet, which shows presence of broad bands ascribed to O···H···N vibrations.

### Refinement

All H atoms were localized from difference Fourier maps. Subsequently, the H atoms bonded to C atoms were included in the model using the riding model approximation with *U*_iso_ set at 1.2 *U*_eq_(parent atom). The H12 atom bonded to the O12 atom was kept using AFIX 147 restraint with *U*_iso_ set at 1.2 *U*_eq_ (parent atom). The H1 atom (participating in strong O···H···N hydrogen bond) *U*_eq_ was refined isotropically and was localized at the centre of the O···N distance. On an examination of a difference Fourier map, the structure was re-refined with this H1 atom disordered over two positions (H1A and H1B): one position nearer to the O11 atom and one position nearer to the N1 atom. For both components the standard O—H and N—H distances were fixed accordingly. The final refined occupancy factors are 0.59 (3) and 0.41 for the major (H1A) and for the minor (H1B) component, respectively. An examination of the resulting difference Fourier map showed that the highest peak of 0.48 e Å^-3^ was located 1 Å from the C21A atom. This was interpreted as slight disorder of the methylene moiety, i.e. over two positions. The disorder was modelled imposing soft SADI restraints (with the allowed deviation of 0.02) on the C–C bond lengths in both components. The positions of the C31 and C11 atoms, bonded to the higher–occupancy C21A component and to the lower–occupancy C21B component were assumed to be the same for both components. The final refined occupancy factors are 0.940 (6) and 0.060 for the higher- and lower-occupancy components, respectively.

### Crystal data

**Table:**

C_10_H_8_N_2_·C_8_H_10_O_8_	*Z* = 1
*M**_r_* = 390.34	*F*_000_ = 204
Triclinic, *P*1	*D*_x_ = 1.537 Mg m^−^^3^
Hall symbol: -P 1	Mo *K*α radiation λ = 0.71073 Å
*a* = 5.642 (4) Å	Cell parameters from 3002 reflections
*b* = 6.966 (4) Å	θ = 2–35º
*c* = 11.680 (8) Å	µ = 0.12 mm^−^^1^
α = 73.55 (5)º	*T* = 100 (2) K
β = 81.34 (5)º	Plate, colourless
γ = 73.85 (5)º	0.40 × 0.18 × 0.04 mm
*V* = 421.6 (5) Å^3^	

### Data collection

**Table:**

Oxford Diffraction KM-4-CCD diffractometer	1034 reflections with *I* > 2σ(*I*)
Radiation source: fine-focus sealed tube	*R*_int_ = 0.030
Monochromator: graphite	θ_max_ = 28.4º
*T* = 100(2) K	θ_min_ = 3.2º
ω scans	*h* = −7→7
Absorption correction: none	*k* = −7→9
3650 measured reflections	*l* = −15→15
1946 independent reflections	

### Refinement

**Table:**

Refinement on *F*^2^	Secondary atom site location: difference Fourier map
Least-squares matrix: full	Hydrogen site location: inferred from neighbouring sites
*R*[*F*^2^ > 2σ(*F*^2^)] = 0.045	H-atom parameters constrained
*wR*(*F*^2^) = 0.083	*w* = 1/[σ^2^(*F*_o_^2^) + (0.027*P*)^2^] where *P* = (*F*_o_^2^ + 2*F*_c_^2^)/3
*S* = 1.01	(Δ/σ)_max_ < 0.001
1946 reflections	Δρ_max_ = 0.30 e Å^−^^3^
135 parameters	Δρ_min_ = −0.19 e Å^−^^3^
2 restraints	Extinction correction: none
Primary atom site location: structure-invariant direct methods	

### Fractional atomic coordinates and isotropic or equivalent isotropic displacement parameters (Å^2^)

**Table:**

	*x*	*y*	*z*	*U*_iso_*/*U*_eq_	Occ. (<1)
O11	0.4836 (2)	0.4390 (2)	0.68732 (11)	0.0338 (4)	
H1A	0.6275	0.3764	0.6676	0.051*	0.59 (3)
N1	0.9093 (3)	0.2488 (2)	0.61058 (14)	0.0231 (4)	
H1B	0.7625	0.3105	0.6381	0.035*	0.41 (3)
C2	1.0784 (3)	0.1253 (3)	0.68554 (17)	0.0265 (5)	
H2	1.0378	0.1051	0.7693	0.032*	
C3	1.3105 (3)	0.0261 (3)	0.64525 (16)	0.0272 (5)	
H3	1.4260	−0.0608	0.7012	0.033*	
C4	1.3754 (3)	0.0531 (3)	0.52342 (16)	0.0208 (4)	
C5	1.1967 (3)	0.1826 (3)	0.44570 (16)	0.0232 (5)	
H5	1.2318	0.2059	0.3615	0.028*	
C6	0.9677 (3)	0.2764 (3)	0.49322 (16)	0.0237 (5)	
H6	0.8475	0.3640	0.4398	0.028*	
C11	0.1384 (3)	0.4629 (3)	0.97960 (16)	0.0276 (5)	
H11	0.2287	0.5506	1.0016	0.033*	
C21A	0.1846 (4)	0.4914 (3)	0.84558 (17)	0.0250 (7)	0.940 (6)
H21A	0.1416	0.6412	0.8062	0.030*	0.940 (6)
H21B	0.0719	0.4285	0.8193	0.030*	0.940 (6)
C31	0.4490 (4)	0.3983 (3)	0.80273 (18)	0.0271 (5)	
O21	0.6106 (2)	0.29297 (19)	0.87162 (11)	0.0272 (3)	
C12	0.2377 (3)	0.2422 (3)	1.05080 (18)	0.0262 (5)	
O12	0.1933 (2)	0.1051 (2)	1.00261 (12)	0.0316 (4)	
H12	0.2479	−0.0148	1.0450	0.047*	
O22	0.3354 (2)	0.1971 (2)	1.14272 (12)	0.0387 (4)	
C21B	0.290 (6)	0.552 (3)	0.875 (2)	0.014 (9)*	0.060 (6)
H21C	0.4014	0.6144	0.9028	0.017*	0.060 (6)
H21D	0.1785	0.6655	0.8207	0.017*	0.060 (6)

### Atomic displacement parameters (Å^2^)

**Table:**

	*U*^11^	*U*^22^	*U*^33^	*U*^12^	*U*^13^	*U*^23^
O11	0.0236 (8)	0.0379 (10)	0.0249 (9)	0.0087 (7)	0.0034 (6)	−0.0030 (7)
N1	0.0167 (9)	0.0238 (10)	0.0249 (10)	0.0016 (7)	0.0005 (7)	−0.0074 (8)
C2	0.0243 (11)	0.0321 (12)	0.0190 (11)	−0.0009 (10)	0.0012 (9)	−0.0073 (9)
C3	0.0207 (11)	0.0308 (12)	0.0233 (12)	0.0025 (9)	−0.0028 (9)	−0.0042 (10)
C4	0.0178 (10)	0.0214 (11)	0.0220 (11)	−0.0027 (8)	0.0003 (9)	−0.0066 (9)
C5	0.0242 (11)	0.0228 (11)	0.0191 (11)	−0.0023 (9)	0.0016 (9)	−0.0051 (9)
C6	0.0226 (11)	0.0224 (11)	0.0202 (11)	0.0001 (9)	−0.0043 (9)	−0.0006 (9)
C11	0.0227 (11)	0.0240 (12)	0.0251 (11)	0.0066 (9)	0.0032 (9)	−0.0036 (9)
C21A	0.0237 (13)	0.0196 (13)	0.0236 (13)	0.0035 (10)	−0.0008 (10)	−0.0021 (10)
C31	0.0285 (12)	0.0209 (12)	0.0264 (12)	−0.0009 (10)	0.0038 (10)	−0.0056 (10)
O21	0.0232 (7)	0.0243 (8)	0.0267 (8)	0.0026 (6)	−0.0017 (7)	−0.0035 (7)
C12	0.0179 (11)	0.0265 (12)	0.0268 (12)	0.0039 (9)	0.0072 (9)	−0.0088 (10)
O12	0.0290 (8)	0.0227 (8)	0.0353 (9)	0.0008 (7)	−0.0042 (7)	−0.0015 (7)
O22	0.0421 (9)	0.0361 (9)	0.0264 (8)	0.0121 (7)	−0.0098 (7)	−0.0076 (7)

### Geometric parameters (Å, °)

**Table:**

N1—C6	1.332 (2)	C4—C5	1.400 (3)
N1—C2	1.336 (2)	C4—C4^i^	1.497 (4)
C31—O11	1.293 (3)	C5—C6	1.387 (3)
C31—O21	1.237 (2)	C5—H5	0.95
C12—O12	1.331 (2)	C6—H6	0.95
C12—O22	1.202 (2)	C11—C21A	1.512 (3)
O11—C31	1.293 (2)	C11—C12	1.519 (3)
O11—H1A	0.84	C11—C11^ii^	1.549 (4)
N1—H1B	0.88	C11—H11	1.00
C2—C3	1.382 (3)	C21A—C31	1.523 (3)
C2—H2	0.95	C21A—H21A	0.99
C3—C4	1.387 (3)	C21A—H21B	0.99
C3—H3	0.95	O12—H12	0.84
			
C6—N1—C2	118.6 (2)	C4—C5—H5	120.4
C21A—C11—C12	113.4 (2)	N1—C6—C5	122.73 (17)
C21A—C11—C11^ii^	113.0 (2)	N1—C6—H6	118.6
O21—C31—O11	124.2 (2)	C5—C6—H6	118.6
O22—C12—O12	124.0 (2)	C12—C11—C11^ii^	109.00 (19)
C31—O11—H1A	109.5	C21A—C11—H11	107.0
C6—N1—H1B	120.7	C12—C11—H11	107.0
C2—N1—H1B	120.7	C11^ii^—C11—H11	107.0
N1—C2—C3	122.18 (18)	C11—C21A—C31	114.83 (18)
N1—C2—H2	118.9	C11—C21A—H21A	108.6
C3—C2—H2	118.9	C31—C21A—H21A	108.6
C2—C3—C4	120.20 (18)	C11—C21A—H21B	108.6
C2—C3—H3	119.9	C31—C21A—H21B	108.6
C4—C3—H3	119.9	H21A—C21A—H21B	107.5
C3—C4—C5	117.12 (17)	O21—C31—C21A	123.24 (18)
C3—C4—C4^i^	121.7 (2)	O11—C31—C21A	112.54 (18)
C5—C4—C4^i^	121.2 (2)	O22—C12—C11	123.9 (2)
C6—C5—C4	119.19 (17)	O12—C12—C11	112.07 (18)
C6—C5—H5	120.4	C12—O12—H12	109.5
			
C6—N1—C2—C3	0.0 (3)	C11^ii^—C11—C21A—C31	−173.3 (2)
N1—C2—C3—C4	0.1 (3)	C11—C21A—C31—O21	5.5 (3)
C2—C3—C4—C5	−0.2 (3)	C11—C21A—C31—O11	−175.45 (18)
C2—C3—C4—C4^i^	−179.6 (2)	C21A—C11—C12—O22	141.0 (2)
C3—C4—C5—C6	0.1 (3)	C11^ii^—C11—C12—O22	−92.2 (3)
C4^i^—C4—C5—C6	179.53 (19)	C21A—C11—C12—O12	−42.0 (2)
C2—N1—C6—C5	−0.1 (3)	C11^ii^—C11—C12—O12	84.8 (2)
C4—C5—C6—N1	0.0 (3)	C5—C4—C4^i^—C3^i^	0.6 (4)
C12—C11—C21A—C31	−48.6 (3)		

Symmetry codes: (i) −*x*+3, −*y*, −*z*+1; (ii) −*x*, −*y*+1, −*z*+2.

### Hydrogen-bond geometry (Å, °)

**Table:**

*D*—H···*A*	*D*—H	H···*A*	*D*···*A*	*D*—H···*A*
O11—H1A···N1	0.84	1.73	2.565 (3)	173
N1—H1B···O11	0.88	1.69	2.565 (3)	177
O12—H12···O21^iii^	0.84	1.91	2.747 (3)	175

Symmetry codes: (iii) −*x*+1, −*y*, −*z*+2.
